# The Role of the Innate Immune System in Wear Debris-Induced Inflammatory Peri-Implant Osteolysis in Total Joint Arthroplasty

**DOI:** 10.3390/bioengineering9120764

**Published:** 2022-12-04

**Authors:** John Patrick Connors, John W. Stelzer, Patrick M. Garvin, Ian J. Wellington, Olga Solovyova

**Affiliations:** Department of Orthopaedic Surgery, University of Connecticut, Farmington, CT 06032, USA

**Keywords:** arthroplasty, cytokines, implant, osteolysis, wear debris

## Abstract

Periprosthetic osteolysis remains a leading complication of total hip and knee arthroplasty, often resulting in aseptic loosening of the implant and necessitating revision surgery. Wear-induced particulate debris is the main cause initiating this destructive process. The purpose of this article is to review recent advances in understanding of how wear debris causes osteolysis, and emergent strategies for the avoidance and treatment of this disease. A strong activator of the peri-implant innate immune this debris-induced inflammatory cascade is dictated by macrophage secretion of TNF-α, IL-1, IL-6, and IL-8, and PGE2, leading to peri-implant bone resorption through activation of osteoclasts and inhibition of osteoblasts through several mechanisms, including the RANK/RANKL/OPG pathway. Therapeutic agents against proinflammatory mediators, such as those targeting tumor necrosis factor (TNF), osteoclasts, and sclerostin, have shown promise in reducing peri-implant osteolysis in vitro and in vivo; however, radiographic changes and clinical diagnosis often lag considerably behind the initiation of osteolysis, making timely treatment difficult. Considerable efforts are underway to develop such diagnostic tools, therapies, and identify novel targets for therapeutic intervention.

## 1. Introduction

**Figure 1:**
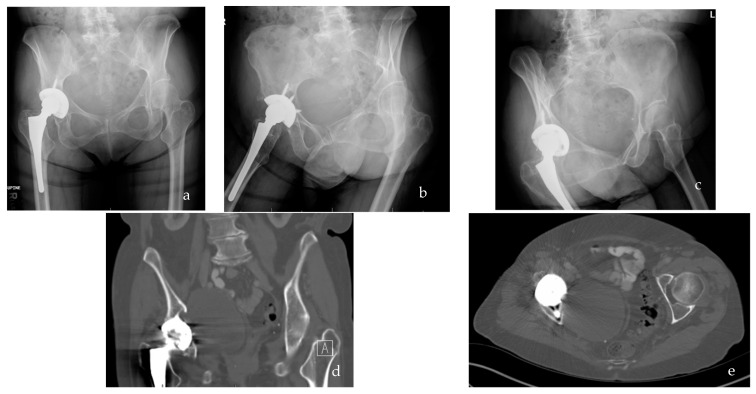
(**a**–**c**) AP pelvis and Judet radiograph demonstrating late-stage osteolysis of the right total hip arthroplasty as evidenced by lucency around the implant-bone interface of the acetabular cup. (**d**,**e**) Coronal and axial computed tomography (CT) demonstrating late-stage osteolysis of the rigt total hip arthroplasty as evidenced by lucency throughout acetabular cup periphery, and ischium.

As the population ages, there is an ever-increasing frequency of total joint arthroplasty, with an estimated prevalence of 4.5 million and 6.7 million people living with a prosthetic hip or knee, respectively [1]. While improvements in surgical technique and materials used are increasing the longevity of arthroplasties, revision surgery will continue to be a necessary proportion of joint replacement [2,3]. At present, revision rates for osteolysis for total hip (THA) and total knee arthroplasty (TKA) range between 3.5 and and 5% of all knee and hip revisions, respectively [4,5,6,7]. While representing a small percentage of revisions, the lack of available bone stock and reduced muscular function (Figure 1) portends need for increasingly complex revisions including additional augments, stability, and constraint exposing patients to increased risk of serious, long-term complications including infection and instability [8]. Therefore, diagnosing and preventing debris wear-induced osteolysis are important targets to improve long-term outcomes of total joint arthroplasty.

**Figure 2:**
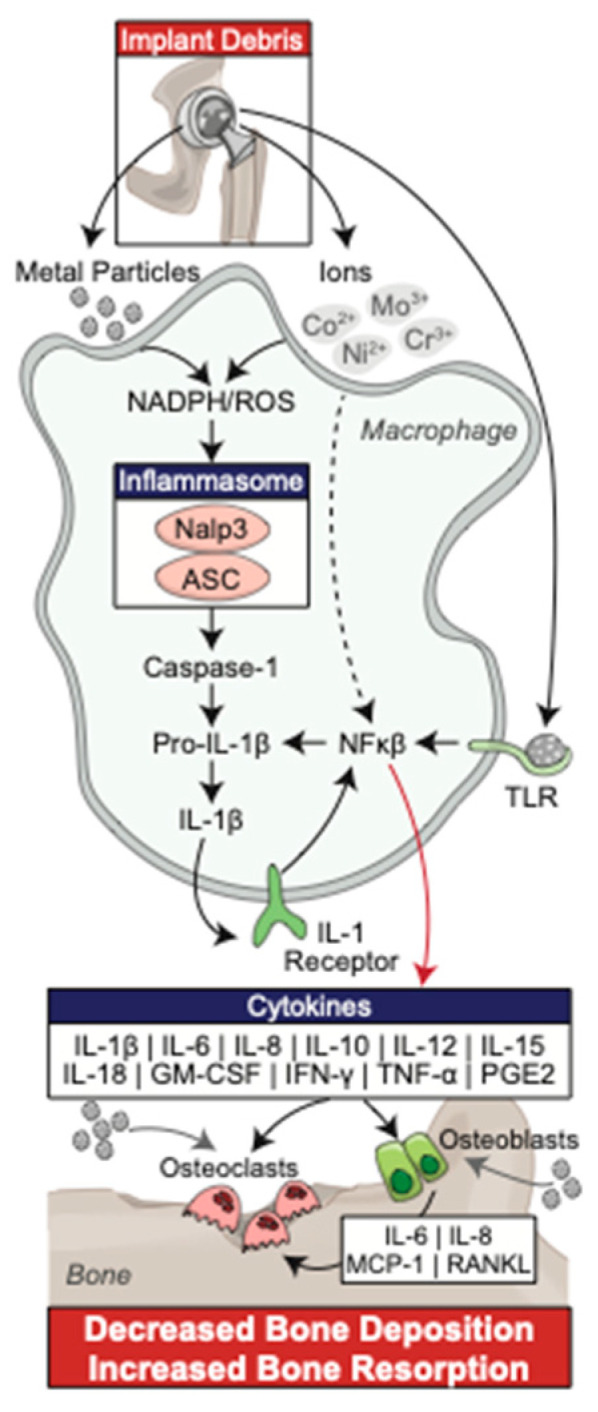
Schematic of the inflammasome pathway and its central role in the development of wear debris associated peri-implant osteolysis.

Mechanical failure due to aseptic loosening was historically the most common long-term complication of total joint arthroplasty in conventional polyethylene implants, and despite the use of highly cross-linked polyethene is still responsible for the many of revision surgeries today in both hip and knee arthroplasty at greater than one year post operation [3,9]. Although various factors may play a role in aseptic loosing, including implant wear properties and host related factors, the formation of implant wear-related polyethylene debris particles have been described in the literature as a fundamental aspect of this bony destruction [10,11,12,13,14]. This localized bone destruction, known as peri-implant osteolysis (PPOL), is initiated by the phagocytosis of small wear debris particles by cells of the monocyte/macrophage cell lineage, leading to their proliferation, differentiation, and activation (Figure 2) [15]. These events lead to upregulation of intracellular signal transduction involving the transcription activation of NFκB, nuclear translocation, and upregulation of gene expression. This triggers increased cytokine and chemokine production, leading to a pro-inflammatory state, which disrupts the homeostatic balance between bone formation and resorption, ultimately promoting osteolysis [16,17,18]. This review summarizes the current literature regarding the innate immune response to wear debris particles and the subsequent induction of osteolysis, as well as immunomodulation for the prevention and treatment of osteolysis in high-risk patients.

## 2. Innate Immune Response to Wear Debris Particles

### 2.1. Macrophages

The initial inflammatory response to wear debris has historically been understood as a product of macrophage reactivity; therefore, a primary focus of investigation has been targeted at the reduction in osteolysis. Rao et al. demonstrated a predominance of M1 macrophages in synovium and pseudo-membrane in patients undergoing revision total hip or knee arthroplasty with radiographic evidence of osteolysis, as well as an in an in vitro murine model in response to intra-articular challenge with wear debris [19]. The normal cytokine profile of M1 and M2 macrophages differ significantly. M1 macrophages induce a pro-inflammatory state with production of mediators including TNF-α, IL-1, IL-6, IL-12, IL-23, granulocyte-macrophage colony stimulating factor (GM-CSF), macrophage colony stimulating factor (M-CSF), platelet derived growth factor, and epidermal growth factor, as well as increased expression of iNOS and HLA-DR. Alternatively, M2 macrophages produce a milieu that induces bone healing, debris scavenging, wound healing, and angiogenesis through production of mediators such as IL-4, IL-10, IL-13, and increased expression of CCL1, CCL18, mammalian chitinase Ym1, Arginase 1, and FIZZ1.

The induced polarization to the M1 phenotype has significant clinical value as both a target for drug therapy and more simply, as an indicator of the biologically active role of this wear debris [20,21,22]. The complete cytokine profile of the M1 macrophage response has not yet been fully elucidated, and it is expected that more obscure and occult cytokines and tissue responses are involved in this reactivity as well [15]. While M-CSF activates osteoclasts directly; IL-1, TNF-α, and IL-6 have been shown to influence osteoblasts and other cells that direct the formation of osteoclasts. Additionally, GM-CSF directly regulates the formation of multinucleated giant cells, which act in a mechanism analogous to osteoclastic destruction of bone [23]. While the complete mechanism is not yet fully understood, Figure 2 demonstrates the central role of the milieu of M1 cytokines, known as the inflammasome pathway, in the biologic response to wear debris with subsequent development of osteolysis [15,23]. 

### 2.2. Toll-like Receptors

Chemokine expression by peri-implant cells, including macrophages, fibroblasts, and osteoblasts, exposed to wear debris plays a central role in the innate immune response leading to osteolysis. Although the periprosthetic inflammatory pathway described above has been well studied, less is known about the mechanisms of the initial interaction between wear debris and the phagocytic cells responsible for initiation of the inflammasome. A study by Gu et al. examined the literature regarding Toll-like receptors (TLRs) and chemokines in the activation of macrophages in the induction of the osteolysis inflammasome pathway [24]. TLRs belong to a class of pattern recognition receptors that enable the innate immune system to distinguish between self and non-self structures, and can recognize a myriad of stimuli, including endogenous damage associated molecular patterns (DAMPs) and exogenous pathogen associated molecular patterns (PAMPs) [25,26]. Over 13 individual mammalian TLRs have been identified, with TLR2 and TLR4 most commonly associated with aseptic loosening [25]. A study by Greenfield et al. found that TLR2-/- knockout mice had reduced radiographic evidence of osteolysis when compared to wild-type mice in vivo, and that TLR2 -/- knockout murine macrophages secreted reduced TNF-α when challenged with titanium wear debris, all suggestive of TLR2 playing a critical role in aseptic loosening [27]. Similar findings have been identified in TLR4 receptors, which typically recognize LPS, mannan, glycoinositol phospholipids, envelope proteins, or self-proteins including heat shock proteins (HSP) 60 and 70 [28,29]. Initial interest in TLR4 as a mediator of aseptic loosening began as increased expression of the receptor was identified in in tissue surrounding explanted prostheses due to osteolysis by Takagi in 2007 [30]. Subsequent investigation identified decreased osteolysis in knockout TLR4-/- mice, as well as an inhibited inflammatory response when exposed to wear particles, as comparted to wild type TLR4 mice [31]. Several groups also examined the role of particle composition as it effects TLR4 activation. Ultra-high molecular weight polyethylene (UHMWPE), a common weight bearing surface in total hip and knee replacements, was found to upregulate expression of TLR4 and HSP60 receptors on monocytes, leading to downstream upregulation of pro-inflammatory cytokines [32]. 

**Figure 3:**
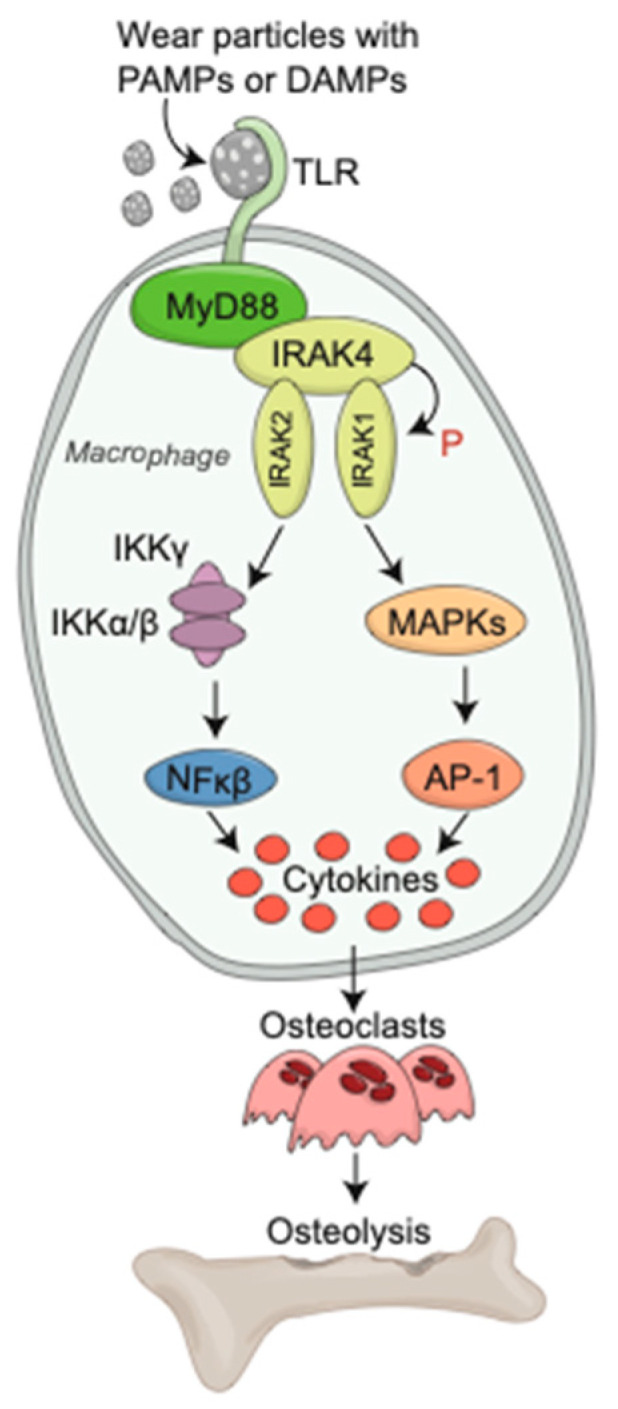
Wear debris particles induce the TLR signaling cascade. TLRs recognize wear particles with adherent PAMPs or DAMPs via MyD88. The binding of TLR and MyD88 phosphorylates IRAK4 which subsequently phosphorylates IRAK1. The activation of AP-1 and NF-κB leads inflammatory cytokine production and osteoclast differentiation and maturation, leading to osteolysis.

Titanium, like UHMWPE, is a common implant used in orthopaedic reconstruction, and also generates debris leading to aseptic loosening. A similar upregulation in cytokine production and osteolysis was found with exposure to titanium particles coated with lipopolysaccharide (LPS) through binding to TLR4 [33]. It remains unclear, however, whether an endotoxin coating, such as LPS, is required for TLR4 activation. Takagi et al. demonstrated that LPS exposure downregulates TLR4 mRNA expression, likely due to an auto or paracrine inflammatory cytokine downregulation as a protective mechanism to avoid damage caused by excessive inflammatory response [30]. Given this strong evidence supporting the role of TLR2 and TLR4 in the activation of the inflammasome pathway, their activation and downstream effects has been a target of much research. Several studies have been influential in identifying the molecular recruitment and downstream effects of wear particles and can be seen in Figure 3, which illustrates wear particles binding to TLR2/4, with adherent PAMPs or DAMPs. With the binding of wear particles to TLR, there is subsequent rearrangement of tyrosine inducible regions and adaptor protein recruitment, including MyD88, TIRF, and TIRAP. Activation of MyD88 phosphorylates IRAK4, which subsequently phosphorylates IRAK1. This subsequently leads to downstream activation of AP-1 and NFκB, leading to nuclear translocation and upregulation of inflammatory cytokine production. This plays a pivotal role in the induction of osteoclast differentiation and upregulation, leading to osteolysis [26,34]. 

### 2.3. Chemokines and Chemokine Receptors

As discussed above, chemokine expression by peri-implant cells, including macrophages, fibroblasts, and osteoblasts, that are exposed to wear debris also plays a central role in the innate immune response leading to osteolysis [24]. Chemokines are a class of small proteins that play a critical role in normal leukocyte activation and migration. The intracellular effects of these chemokines are mediated by a family of G-protein coupled receptors on effector cell surfaces, and are classified into the following four groups based on the class of chemokine that they bind: CXCR, CCR, CX3R, and XCR. [35,36] Given their central role in activation of the innate immune system, these molecular interactions have been a target of research for better understanding the pathogenesis of osteolysis [37,38]. Recently, an improved understanding of the role of chemokines in debris-induced cellular migration has been elicited. It was historically understood that wear debris particles stimulated only a local response with resident osteoblasts and macrophage/monocytes activated to produce IL-1, IL-6, and TNF-α [23]. However, landmark studies by Lind et al. and Nakashima et al. identified the role of titanium and UHMWPE debris in the induction of chemokine expression in macrophage/monocytes, fibroblasts, and osteoblast leading to cellular migration [39,40]. Additional studies by both Gibon et al. and Ren et al. demonstrated in vivo chemotaxis of macrophages in response to wear particles [41,42,43]. Several key chemokines specific to aseptic loosening pathology have been identified, including IL-8, monocyte chemoattractant protein (MCP)-1, MIP-1a, CCL17/TARC, and CCL22/MDC. 

IL-8, a well-described chemokine, is a member of the CXC class, is classically understood to function as a neutrophil chemotactic factor [38]. Much like its TNF-α, IL-1, and IL-6 counterparts, IL-8 has been identified in high levels in tissue and synovial fluid surrounding failed, aseptic loosened prostheses [44]. As such, IL-8 can be used as a marker for early detection of aseptic loosening in patients with continued pain without radiographic evidence of loosening. A study by Kaufman et al. utilized protein arrays to identify macrophage/monocyte cytokine production to wear debris particle challenge in vitro, utilizing UHMWPE, TiAlV, Cobalt Chrome (CoCr), and alumina wear debris. They identified that IL-8 was produced in human macrophages in vitro at 5–900 times greater than a challenge with normal saline, and that TiAlV particles were the most stimulatory, with UHWPE and CoCr stimulating increased IL-8 production, though dampened compared to the titanium alloy [44,45]. A recent study by Haleem-Smith et al. identified upregulated IL-8 production by mesenchymal stem cells exposed to Ti, UHMWPE, and CoCr [46]. This increased expression leads to migration of effector cells, including those of the macrophage/monocyte lineage and osteoclasts to the periprosthetic region, leading to increased osteolysis. MCP-1, MIP1 (CCL-2), and MIP1a have all also been identified in periprosthetic tissue surrounding failed, aseptically loosened implants. These chemokines are upregulated in vitro after macrophage exposure to various wear debris particles, as demonstrated by Nakashima et al [40]. Several studies have also evaluated the role of MIP1(CCL-2) through either CCR2 receptor blockade or in a CCR2 Receptor -/- knockout mouse model, identifying elimination of migration of MSCs in vitro [41,47]. Blockade of CCR4 on osteoclast precursors, the conjugate receptor for chemokines CCL17/TARC and CCL22/MDC expressed on mature osteoclasts, reduced recruitment of osteoclast precursors to the bone-prosthesis interface in an in vitro human model [48]. 

## 3. Bone and Soft Tissue Relationship

### 3.1. Osteoclasts

The bone resorptive function of osteoclasts is central to the pathogenesis of osteolysis. The RANK, RANK ligand (RANK-L), osteoprotegerin (OPG) system is critical to the activation of osteoclasts. Osteoblasts and stromal cells express RANK-L, which subsequently binds to its receptor, RANK, on the surface of osteoclasts and their precursors. This binding upregulates the differentiation of precursors into multinucleated osteoclasts and promotes osteoclast survival. In a mechanism to protect bone from excessive resorption, OPG is secreted by osteoblasts and osteogenic stromal stem cells and binds to RANK, preventing interaction with RANKL and subsequent osteoclast activation. There is significant interplay and crosstalk between the cells participating in the RANK/RANKL/OPG pathway and the innate immune system [49,50]. The degree to which other, non-osteoclast cells, with the potential to resorb bone, are activated by the RANK/RANKL system is unknown, and the complete role of released cytokines, such as TNF-α or IL-1 in activation of osteoclasts, has yet to be fully elucidated [15]. Kadoya et al. demonstrated that multinucleated giant cells express some markers similar to those characteristic of osteoclasts, including tartrate-resistant acid phosphatase (TRAP) and vitronectin receptor, though this was identified only on the bone side of the bone-implant interface, it was not identified in macrophages localized to the implant surface [49]. Further in vitro studies have demonstrated that macrophages localized to the hip capsule, distinguished from their osteoclast counterparts both by antigenic phenotype and lack of response to calcitonin, are themselves capable of mild bone resorption after exposure to wear debris particles [51]. However, despite these findings, if the bone resorbing capabilities of macrophages is comparable to that of osteoclasts, given their ubiquity and near ontogenic relationship with osteoclasts, it remains to be determined if macrophages directly play a role in osteolysis [15]. The inverse has also been evaluated in several studies. Wang et al. identified the capability of osteoclasts to phagocytose wear debris particles of various materials, including ceramic, metallic, and polymers. They identified that these osteoclasts maintained full functional abilities after the phagocytosis of such wear debris particles demonstrating that, at least in vitro, there is substantial plasticity between these cell types involved in osteolysis deriving from the same precursor cells in the bone marrow [52,53]. As discussed previously, Haleem-Smith et al. identified the ability of mesenchymal stem cells, precursors for both macrophage/monocytes and osteoclasts, to participate in the formation of implant associated osteolysis, with phagocytosis of wear particles leading to decreased proliferation and increased production of pro-inflammatory cytokine IL-8 [46]. 

### 3.2. Osteoblasts

As the stimulatory cells in the RANK/RANKL/OPG pathway, osteoblasts play a key role in the natural homeostasis of bone and have been well described as critical players in the development of wear debris mediated osteolysis. Both Vermes et al. and Zreiqat et al. initially identified the adverse effects of wear debris material on the formation and function of osteoblasts [54,55]. Subsequently, titanium particles were identified as a downregulator of gene expression of type I collagen by osteoblasts, and UHMWPE particles were identified to alter differentiation of osteoblast precursors [56,57]. Pioletti et al. first identified the cytotoxic effects of wear debris particles on osteoblasts, as well as the alteration of fibronectin gene expression and cellular adhesion strength [58,59]. Most recently, Pioletti and Kottelat evaluated the role of osteoblasts in the altered cytokine and chemokine milieu through exposure to wear debris particles. They utilized primary human osteoblasts exposed to titanium particles in vitro, then utilized RT-PCR to measure relative gene expression when compared to non-exposed controls. They identified that when compared to unexposed human osteoblasts, the titanium exposed cells demonstrated increased production of osteoclastogenic factors RANK-L and M-CSF, and pro-inflammatory cytokines IL-6 and IL-8. They also identified increased expression of VEGF and matrix metalloprotease(MMP)-1, and decreased expression of type I collagen RNA [60]. These findings are highly suggestive of the importance of osteoblasts as critical mediators in the development of wear debris induced osteolysis.

### 3.3. Fibroblasts

As the predominant cell type at the bone-prosthesis interface, and as mediators of the development and differentiation of osteoclasts, fibroblasts have long been thought to play a role in the modulation of normal bone homeostasis and the development of wear debris mediated osteolysis [61]. Koreny et al. evaluated the role of fibroblasts and fibroblast-derived factors in the mediation of peri-implant osteolysis. Fibroblasts were stimulated in vitro by applying pro-inflammatory cytokines to cultured media, obtained from explanted synovial tissue from osteolytic knee and ankle implants, peri-prosthetic interface membrane, and titanium wear debris particles. RNase protection assays were then used to identify altered gene expression in the exposure groups, and ELISA, Western blot hybridization, and flow cytometry were used to determine changes in fibroblast protein expression. They identified elevated levels of TNF-α, MCP-1, IL-1, IL6, IL-8, and VEGF in the conditioned media from explanted interface membranes. Fibroblasts were found to phagocytose particulate wear debris, leading to subsequent upregulation of previously discussed inflammasome cytokines and chemokines, including MMP-1, MPC-1, IL-1, IL-6, IL-8, COX-1, COX-2, leukemia inhibitory factor, and TGFβ1/TGFβ1 receptor. Additionally, when these interface membrane fibroblasts were cultured with media with containing TNF-α or IL-1, they demonstrated upregulation of RANKL and OPG. Furthermore, when these stimulated fibroblasts were cocultured with bone marrow aspirate in the presence of macrophage colony stimulating factor (M-CSF), staining with tartrate resistant acid phosphatase (TRAP) identified induction of osteoclastogenesis [62]. These findings suggest that interface fibroblasts directly respond to wear debris particulates, potentially through direct phagocytosis. This leads to upregulation of the inflammasome and may be actively involved in the induction of osteoclastogenesis and activation of mature osteoclasts, potentially playing a critical role in the development of periprosthetic bone destruction.

## 4. Therapeutic Targets of Aseptic Loosening through Modulation of the Innate Immune Response

At present, there are no established alternatives to revision surgery for PPOL. While anti-resorptive have been shown in small studies to provide some moderate benefit, these benefits have not been supported in randomized trials. With the recent development and applications of biologic drugs as potential therapies for various pathologic processes in medicine, there are immense opportunities for immunomodulation as a means to reduce or prevent the need for revision surgery for osteolysis or aseptic loosening. In this section, we review the current clinically validated immunomodulatory treatments of osteolysis, as well as novel targets identified in both human and translational studies.

Non-biologic immunomodulators, namely bisphosphonate treatment, have historically been thought to play a potential role in PPOL. Bisphosphonates remain the most widely used agent to treat osteoclast-driven bone disease, including osteoporosis and Paget’s disease, and have been historically used to theoretically increase the clinical survivorship and decrease the rate of revision surgery though modulation of osteoclast activity [63]. Functioning largely based on their chemical structure, osteoclasts can be grouped into two distinct categories, those being nitrogen containing and non-nitrogen containing [64,65]. Preferentially incorporated into bone mineral due to their high affinity for hydroxyapatite, the first developed bisphosphonate medications, possessed R^2^ side chains lacking nitrogen, and given their structural similarity to inorganic phosphate, were incorporated into newly formed adenosine triphosphate (ATP) by class II aminoacyl-transfer RNA synthetases after osteoclast mediated uptake from mineralized bone. Subsequent intracellular accumulation of non-hydrolyazable ATP analogues is thought to be cytotoxic to osteoclasts, inhibiting multiple ATP dependent processes, and leading to osteoclast apoptosis. In contrast, later generation bisphosphonates possessed R^2^ side chains containing nitrogen, and their mechanism of osteoclast cytoxicity is distinctly different from that of their early-generation counterparts. These nitrogen containing bisphosphonates bind to and inhibit the activity of farnesyl pyrophosphate synthase, a key regulator in the mevalonic acid pathway critical to the production of cholesterol and other sterols. As such, the post-translational modification of proteins is inhibited, leading to downstream cellular apoptosis. While this is non-selective for simply osteoclasts, the high-affinity of nitrogen containing bisphosphonates for hydroxyapatite before osteoclast endocytosis allows for selective inactivation of osteoclasts specifically [64,65,66]. In the setting of TJA, the primary concerns with the use of bisphosphonates involve the role of osteoclast inhibition in at the bone-implant interface in regard to both biologic ingrowth of porous surfaces as well as reducing the effect of wear debris on the histologic stability of the bone implant interface [63]. Numerous clinical studies have evaluated the role for bisphosphonate supplementation, identifying increased radiologic bony ingrowth [67,68], as well as decreased rate of revision for aseptic loosening [69], and decreased all cause revision rate with bisphosphonate used for the first year following arthroplasty [70,71], though these studies are limited by small sample sizes and failure to evaluate patients following the development of osteolytic defects. Von Knoch et al. in 2007 examined the utility of bisphosphonates in a leporine model of particulate wear at 6 and 12 weeks’ post-implantation. Their group identified both a significant histologic and radiographic increase in bone thickness at 6 and 12 weeks with administration of both alendronate and zolendronate in both the minimal wear and high wear leporine knees. Further histologic analysis demonstrates increased bone volume in bisphosphonate treated knees, independent of the presence of wear particles, suggesting that bisphosphonates may provide protective effect against PPOL [72]. However, these findings have not been supported in randomized clinical trials, as Rubash et al. randomized 123 patients with established osteolytic lesions, finding that daily oral administration of both low and high dose alendronate did not affect change in radiological size, visual analog scale pain scores, or risk of progression to revision surgery over the 18-month study period when compared to placebo [73]. Further clinical trials are necessary to elucidate the benefits of bisphosphonates in prevention and treatment of PPOL, particularly in the treatment of previously developed osteolytic defect.

At this juncture, the anti-RANKL biologic denosumab is considered the most realistic option for prevention of osteolysis, due to its known mechanism of action, superior efficacy over other drugs, and its limited side effect profile [74,75,76]. Studies in murine models of periprosthetic have previously demonstrated the significant protection against bone resorption at the implant interface with administration of anti-RANKL antibodies, with additional decreases in serum levels of the osteolytic enzyme Tartrate Resistant Acid Phosphatase (TRAP). Mahtma et al. recently completed a double-blind randomized controlled clinical trial examining the effect of denosumab on the immunologic characteristics of the bone-implant interface in revision hip arthroplasty for osteolysis. Twenty-two patients were randomized to treatment with a single dose of subcutaneous denosumab versus placebo prior to their revision THA for osteolysis. Bone surface biopsies were taken 8 weeks later at the time of surgery, with a significant decrease (83%) in osteoclast number at the bone-implant interface, as well as osteoclast surface percentage, osteoblast number, and osteoblast surface percentage in the denosumab group when compared to controls [77]. This is the first study to confirm the immunologic basis for clinical efficacy of anti-RANKL antibodies in the reduction in osteolytic activity in human subjects. While first-in-man, this study is fraught with limitations, particularly in that the included patients already indicated for surgery for management of their osteolytic defects, suggesting significant destruction and likely implant instability. As such, the authors did not include the most likely patients to benefit from such a treatment, that being patients with well-functioning, well-fixed arthroplasties with early evidence of osteolysis. Furthermore, this study fails to clearly elucidate a clinical benefit of anti-RANKL antibodies, particularly in this subset of patients, and further studies should seek to further elucidate a substantial clinical benefit of denosumab treatment, particularly in otherwise asymptomatic patients. Skoldenberg et al. initiated a similar double blind, randomized clinical trial in 2015, to evaluate the benefit of Denosumab on PPOL in a group of 20 patients [78]. While enrollment is complete, at the time of this review, the results of such study remain illusory.

Furthermore, developments in understanding of the Wnt-sclerostin pathway as a central regulator of normal homeostasis of bone have provided a potential target for a single agent to function as combination therapy in high-risk patients. Romosozumab, a humanized mouse anti-sclerostin monoclonal antibody was approved for medical use in the United States in 2019 and has since played a critical role in the treatment of both osteoporosis as well as cancer-mediate osteolysis given its unique capabilities as both anti-resorptive and anabolic in bone [79,80,81,82]. Expressed by mature osteocytes, fibroblast like synovial cells, and even myeloma cells directly, sclerostin functions as a key regulator of the canonical Wnt/βcatenin pathway. Wnt/βcatenin signaling is activated by binding of Wnt proteins to receptor complexes composed of frizzled receptors and co-receptors of the low density lipoprotein receptor-related protein (LRP) family, LRP5 and 6. This event stabilizes βcatenin, induces its translocation to the nucleus, and activates gene transcription. This pathway is known as the canonical Wnt signaling pathway, and controls differentiation of mesenchymal stem cells (MSC) restraining chondrogenic and adipogenic differentiation and favoring osteoblastic differentiation, as well as promoting osteoblast maturation, survival of osteoblasts and osteocytes, and inhibits osteoclast generation by increasing the expression in osteoblasts and osteocytes of OPG. Sclerostin is a potent antagonist of the canonical Wnt pathway, binding to the Wnt Co-receptor LRP5/6, preventing downstream signaling of Wnt. While much has been learned about the role of anti-sclerostin therapy in the setting of quantitative bone loss in osteoporosis and multiple myeloma [74,83], the role of sclerostin in wear-induced PPOL has yet to be elucidated. In a recent study, Jagga et al. in 2021 published on the sclerostin-producing potential of fibroblast-like synoviocytes (FLCs) at the implant host junction. In an in vitro model of FLCs, particular wear upregulated NFκB, COX-2, pro-inflammatory cytokines IL-1β, TNF-α, IL-6, IL-8, IL-11, IL-17, as well as RANKL expression, sufficient to activate osteoclast activity. Inhibition of Wnt signaling was observed in osteoprogenitor cells after the introduction of wear-induced particulate debris, and in the presence of such debris, the stimulation of the canonical Wnt pathway failed to upregulate osteogenic activity, with detection of sclerostin production by FLCs and osteoprogenitor cells. Interestingly, neutralization of SOST in the cell lines partially restored the downregulated Wnt signaling in the presence of wear debris [84]. As such, this study suggests that not only may FLCs play a role in PPOL through the stimulation and upregulation of osteoclast activity, but also through suppression of osteoblastic activity of osteoprogenitor cells, and that inactivation of Sclerostin may play a protective role. While significantly more in vitro and in vivo research is needed to both better elucidate the mechanism of such suppression, as well as translate to animal models and clinical research, the possibilities of such effects are encouraging.

Current biologics indicated for the treatment of rheumatic disease are another potential target, including TNF-α inhibitors such as etanercept, adalimumab, or infliximab, and IL-1 antagonists such as anakinra [84]. While widely used in the treatment of rheumatic disease, there is significant hesitation regarding their application in osteolysis. This is due primarily to the well-established side-effects of drugs targeting the innate and adaptive immune system, leading to immunosuppression, opportunistic infections, and rarely, subtypes of cancer. Additionally, they are associated with a high cost of treatment (~USD 15,000 per year) [84]. Furthermore, off-label utilization of these drugs would require FDA approval. Despite initial interest in the form of a prospective, double-blind, placebo-controlled clinical pilot study, there are no economic models suggesting return on investment necessary to risk time (~12 years) and resources (~USD 800 million) necessary to obtain approval.

Several novel biologics have been evaluated in vitro as potential therapeutic options to arrest osteolysis once detected. One such biologic is AM630, a selective antagonist of cannabinoid receptor (CB) 2 that subsequently inhibits IL-1 and TNF-α. This leads to arrest of the inflammatory reaction previously described leading to osteolysis both in vitro and in vivo in a murine model [85]. LY294002, a PI3K/AKT selective inhibitor, has been found to decrease expression of TNF-α mRNA and downstream protein product in macrophages pretreated with the drug after stimulation with wear particle debris [86]. Tetracycline was also evaluated as a potential treatment given its known inhibition of MMP-9. A group using an in vivo murine model of osteolysis found that despite the MMP-9 inhibitory properties, there was no alteration in MMP-9 or TNF-α expression, but instead highly downregulated gene expression of RANK and RANKL and inhibited wear debris induced osteolysis [84].

## 5. Biomarkers

Finally, while there have been several potential immunologic based targets to prevent osteolysis, clinically validated biomarkers for early detection of prosthesis loosening are critical in the application of such treatments. The natural history of osteolysis is such that once the clinical or radiographic evidence of osteolysis is apparent, the implant may be mechanically loose. Even if medical therapy were to arrest wear debris induced inflammatory osteolysis upon detection, continued micromotion and instability of implants prevents reintegration of the implant [87] While therapeutic targets are of research interest, there is little clinical application until the development of a clinically validated biomarker enables the detection of osteolysis prior to clinical or radiographic presentation, to the development of micromotion and mechanical instability, and prior to the development of severe bone loss requiring augmented reconstruction during revision surgery. Ross et al. examined 24 h urine samples of 26 patients who had previously undergone TJA, with 10 having subsequently developed PPOL, and the remaining 16 serving as healthy controls. Seven candidate biomarkers were measured, including free deoxypyridinoline (DPD), cross-linked N-telopeptides (NTX), interleukin-6 (IL-6), interleukin-8 (IL-8), osteoprotegerin (OPG), α-crosslaps (α-CTX), and β-crosslaps (β-CTX). As an individual biomarker, DPD demonstrated the highest ability to predict osteolysis, with an area under the curve (AUC) in Receiver Operating Characteristic (ROC) analyses of 0.844 at 6 years prior to diagnosis. A panel of α-CTX and IL-6 was able to identify at-risk patients with an AUC of 0.941 or greater at all post-operative time points and an AUC of 1.000 pre-operatively, suggesting the role for such biomarkers in the detection of pre-clinical PPOL [88]. In the only other series in the literature, Li et al. found that biomarker levels, specifically osteocalcin and cross-linked carboxyterminal telopeptide of type I collagen (ICTP), were elevated before implant migration as assessed by radiosteriometric analysis (RSA) in their cohort of 40 TKAs [89]. Further research is needed to identify optimal targets, routes, and timing of testing following arthroplasty to monitor for pre-clinical evidence of osteolysis.

## 6. Limitations

As a retrospective review, there are limitations inherent in this article. As this is a broad, complex topic with ever-changing targets, it is certainly possible that studies with differing outcomes were not included. Given the breadth of literature covered, this review is unable to completely detail overall research strategy, inclusion and exclusion criteria, statistical methods, and limitations of each study. As such, readers with a keen interest would be wise to review the individual studies included before changing practice habits.

## 7. Conclusions

The initiation of aseptic osteolysis around prostheses used in total joint arthroplasty is intimately related to the inflammatory reaction that occurs because of wear debris particles that lead to activation of the innate immune system. This debris-induced inflammation is dictated by macrophage secretion of TNF-α, IL-1, IL-6, and IL-8, and PGE2, leading to peri-implant bone resorption through activation of osteoclasts and inhibition of osteoblasts through several mechanisms, including the RANK/RANKL/OPG pathway. With the increasing incidence and prevalence of TJA, there is a growing need for early diagnosis and intervention for wear debris induced inflammation. Recent research has improved our understanding of how sterile implant debris causes downstream immune activation and peri-prosthetic responses, such as the inflammasome signaling pathway. Targeted immunologic therapies, including application of rheumatic drugs such as infliximab, etanercept, and adalimumab have been the focus of much research, and are being investigated as potential pharmacologic agents against PPOL. Denosumab has shown promise in early clinical trials, however, higher power, longer term clinical studies are necessary to better elucidate the roll of anti-RANKL antibody treatment. Furthermore, SOST/Sclerostin, PI3K inhibitors, or tetracyclines are all promising therapeutic modalities that require further in vitro and in vivo literature to elucidate their potential effects. Most importantly, immunologic detection modalities, such as urinary α-CTX, IL-6, DPD, or ICTP, require further study to identify optimal markers, routes, and timing of testing following arthroplasty to monitor for pre-clinical evidence of osteolysis.

## Data Availability

Not applicable.
